# A Novel Model of Schizophrenia Age-of-Onset Data Challenges the Conventional Interpretations of the Discordance in Monozygote Twin Studies

**DOI:** 10.1155/2013/604587

**Published:** 2013-08-21

**Authors:** Ivan Kramer, L. Elliot Hong

**Affiliations:** ^1^Physics Department, University of Maryland Baltimore County, 1000 Hilltop Circle, Catonsville, MD 21250, USA; ^2^Maryland Psychiatric Research Center, University of Maryland School of Medicine, P.O. Box 21247, Baltimore, MD 21228, USA

## Abstract

The relative importance of genetics and the environment in causing schizophrenia is still being debated. Although the high proportion of monozygote cotwins of schizophrenia patients who are discordant suggests that there may be a significant environmental contribution to the development of schizophrenia, this discordance is predicted by an accumulative multimutation model of schizophrenia onset constructed here implying a genetic origin of schizophrenia. In this model, schizophrenics are viewed as having been born with the genetic susceptibility to develop schizophrenia. As susceptible gene carriers age, they randomly accumulate the necessary mutations to cause schizophrenia, the last needed mutation coinciding with disease onset. The mutation model predicts that the concordance rate in monozygote twin studies will monotonically increase with age, theoretically approaching 100% given sufficient longevity. In dizygote cotwins of schizophrenia patients, the model predicts that at least 71% of cotwins are incapable of developing schizophrenia even though every cotwin and their schizophrenic twin shared a similar early environment. The multimutation model is shown to fit all of the monozygote and dizygote concordance rate data of the principle classical twin studies completed before 1970 considered in this paper. Thus, the genetic hypothesis of schizophrenia can be tested by bringing these studies up to date.

## 1. Introduction

Schizophrenia is a brain disease characterized by delusions, hallucinations, and behavioral and functional disturbances. Once the disease develops, most patients' functioning is seriously impaired. Discovering its cause and cure remain some of the biggest challenges to modern medicine and neuroscience. A fundamental debate on the etiology of schizophrenia is the relative importance of genetics and environmental factors in causing the disease. A consistently higher concordance rate of schizophrenia in monozygotic twins than in dizygotic twins supports the genetic hypothesis. By contrast, external environmental factors are thought to contribute to the development of schizophrenia because a significant proportion of monozygotic twins are discordant. However, this inference from this fact is challenged by the mutation model to be developed in this paper.

The high rate of discordance in monozygotic twins (around 50%) is typically credited to environmental factors [1–3]. However, there is also strong evidence to counter this explanation of this discordance. In monozygote twin studies, the risk for schizophrenia in the offspring of the schizophrenic twins is 16.8%, while it is 17.4% in their normal, discordant, cotwins' offspring—virtually identical rates [4]. These results suggest that the genetic susceptibility to develop the disease is carried by both the schizophrenic and the discordant, nonill cotwin and can be transmitted to the next generation with equal probability even when environments are no longer shared as in twin development.

A common misconception in the literature is the mistaken belief that if genes were 100% responsible for schizophrenia, then “when one identical twin had schizophrenia, there would be 100% chance that that the other twin would have it as well.” Perhaps the best way to demonstrate the fallacy of this argument is to consider the physics of radioactive nuclear decay. 

For example, all uranium-238 nuclei are naturally radioactive and decay in multiple steps ending in the stable nucleus lead-206. Despite the fact that uranium-238 nuclei are completely indistinguishable from each other and are considered to be identical particles, about half of a sample of pure U-238 nuclei remains exactly as it was 4.5 billion years ago, while the other half has decayed into lead-206. In physics, each of these decays, or the lack thereof, is considered a random event, having nothing to do with external triggers, and is entirely dictated by the internal physics of the nucleus [5]. This is the analogue of the schizophrenic identical twin data. Although in physics it is accepted that identical radioactive nuclei do not necessarily evolve in tandem in nature, the discordance in schizophrenic identical twins is typically modeled as environmental contributions in genetic epidemiology.

According to the World Health Organization (WHO), schizophrenia occurs in all the countries of the world with a prevalence rate that is very narrowly proscribed (within a factor of about two between the highest and lowest sites). While there is much local evidence of environmental triggers for increasing prevalence in specific regions and times, a meta-analysis of 188 schizophrenia studies, producing a total of 1,721 prevalence estimates drawn from 46 countries, led to the conclusions that the lifetime prevalence rate was around 0.72% worldwide and there was no significant prevalence difference between males and females or between urban, rural, and mixed sites [6]. Such similar prevalences across diverse climatic, economic, and social environments raise serious doubts of the validity of the environmental model of schizophrenia etiology invoked to explain traditional twin and family data. The incidence as a function of age data produced by diseases with known environmental contributions (e.g., many infectious diseases) fluctuates significantly from country to country around the world and from year to year within a country. 

The fact that some monozygote twins susceptible to developing schizophrenia remain discordant at a certain age does not necessarily mean that external environmental factors contribute to the development of the disease. Analogous to a chain of radioactive nuclear decays, a genetically driven random mutation model is constructed here that is shown to successfully fit disparate schizophrenia age-of-onset data from both general population and twin studies.

## 2. The Independent Multimutation Model of Schizophrenia for Singleton Births

In this section, the age-of-onset schizophrenia prevalence function that will be used to fit singleton and twin study data will be derived from a novel multimutation model (MMM). A relatively simple model for the development of schizophrenia assumes that the brain of every person susceptible to the developing this disease must chronically undergo a series of characteristic changes or mutations, numbering *m*, in any order to get it. The last change occurs at the age-of-onset of schizophrenia. Assuming that every change or mutation is independent of all the others, this model will be called the independent mutation model. What role genetic and/or environmental factors play in causing these characteristic changes to the brain is a question that will be discussed later. The male and female data for a given country or region (risk population) will be separately modeled. 

### 2.1. The Age-of-Onset Schizophrenia Distribution Curve in the Multimutation Model

Consider a random sample of a risk population all born in the same year with the susceptibility to develop schizophrenia later in life. The size of the sample population will be denoted by *N*_*s*_, and the cumulative number of people in this sample that has developed schizophrenia by age *t* will be denoted by *N*(*t*). The fraction of this population that has not developed the *i*th mutation at age *t* is given by exp⁡(−*k*_*i*_ *t*), where *k*_*i*_ is defined as the mutation rate (a constant) for the *i*th change or mutation. The meaning of “mutation” here generically refers to any internally driven biological change that contributes to the onset of schizophrenia and does not necessarily refer to a change in genomic sequence. Thus, the fraction of the susceptible population that has developed the *i*th mutation at age *t*, equivalent to the probability of developing this mutation by age *t*, is given by

(1) $$\begin{array}{c} p_{i}\left(t\right)\equiv 1-\exp\left(-k_{i}t\right),\;i=1,2,3,\ldots ,m, \end{array}$$

where the mutation rate *k*_*i*_ is related to the average time *T*_*i*_ necessary for this mutation to occur by *T*_*i*_ = 1/*k*_*i*_. If *m* independent mutations are required for schizophrenia to develop, then the probability that schizophrenia will develop at age *t* in the susceptible population, a quantity to be called the susceptible prevalence, is given by

(2) $$\begin{array}{c} P_{s}\left(t\right)=\frac{N\left(t\right)}{N_{s}}=p_{1}\left(t\right)p_{2}\left(t\right)p_{3}\left(t\right)\cdots p_{m-1}\left(t\right)p_{m}\left(t\right), \end{array}$$

where the values of the *m* mutation rates (constants) *k*_1_, *k*_2_,…, *k*_*m*−1_, *k*_*m*_ are all independent of each other in general. Thus, in this model the mutations can occur in any order, simultaneously, or at completely different times. Notice that the maximum possible value of *P*_*s*_(*t*) is 1.

Now, suppose a total of *N*_0_ infants are born in a given year in a given region or country. If a fraction *f*_*s*_ of this population cohort is susceptible to developing schizophrenia, then the number of people in the cohort that is susceptible to developing the disease is given by *N*_*s*_ = *f*_*s*_ *N*_0_. If the age of the cohort is denoted by *t* (birth is coincident with age *t* = 0), then the number of people in the cohort that have developed schizophrenia by age *t* will be denoted by *N*(*t*).

The *population* prevalence, or schizophrenia risk at age *t*, for the entire population or cohort is therefore given by

(3) P(t)=N(t)N0≡fsPs(t)=fs·p1(t)p2(t)p3(t)⋯pm−1(t)pm(t).

The fraction of the risk population that develops schizophrenia between the ages of *t* and *t* + *dt* is given by *dP*(*t*), so that the *fractional* incident rate is given by

(4) $$\begin{array}{c} \text{IR}\left(t\right)=\frac{dP\left(t\right)}{dt}=f_{s}\frac{dP_{s}\left(t\right)}{dt}\equiv f_{s}\cdot {\text{IR}}_{s}\left(t\right). \end{array}$$

The best fits to schizophrenia data occurred if *m*_1_ = *m* − 1 mutation rates are all equal to the same constant rate *k*_1_, while the remaining one is equal to another rate *k*_2_ ≠ *k*_1_; then the prevalence function in (3) becomes

(5) P(t)=N(t)N0=fsPs(t)=fs[1−exp⁡(−k1t)]m1[1−exp⁡(−k2t)],

where *m* = *m*_1_ + 1. Since this model depends on 4 parameters (*f*_*s*_, *k*_1_, *k*_2_, and *m*), it will be referred to as the 4-parameter model. If it turns out that *k*_2_ = *k*_1_, then the number of parameters in (5) is reduced to 3, and this simplest possible model will be called the 3-parameter model.

The values of the parameters in the prevalence function in (5), or its corresponding incidence function, depend on the values of four fit parameters, *f*_*s*_, *k*_1_, *k*_2_, and *m*_1_ (or, equivalently, *m*), whose values are determined by a least-squares fit to appropriate data. 

If the set of *n* consecutive data-values used in the fit are denoted by {*d*_*i*_}, and if the corresponding model fit-values are denoted by {*x*_*i*_}, then the square of the error of the fit, to be called *chisq* (chi square), is defined as

(6) $$\begin{array}{c} \text{chisq}\equiv \sum_{i=1}^{n}{\left[x_{i}-d_{i}\right]}^{2}. \end{array}$$

The better the fit to the data, the smaller the value of chisq returned by the fit.

### 2.2. Results of Fitting the MMM to Schizophrenia Age-of-Onset Data for Singleton Cases

Unlike point or lifetime prevalence, true age-of-onset prevalence rate in a given population is difficult to ascertain for schizophrenia because the definition of “onset” does not have a common consensus in many cases of insidious onset or prolonged prodromal cases. A relatively objective estimate for age-of-onset is first hospitalization for psychotic break, especially in the earlier era where hospitalization was still widely available and considered a standard of care for the first psychotic episodes in schizophrenia patients. Therefore, we used the schizophrenia age-at-first admission incidence rate data for USA hospitals by Kramer et al. (see Table 3.4 in [7]). These data were compiled for the historical period before or at the beginning of the widespread use of antipsychotic medications. We assume that this first hospital admission incidence rate by age range was proportional to the true age-of-onset by the same age range in the general population. However, the true rate should be higher because a proportion of first onset cases was assumed not hospitalized.

**Figure 1:**
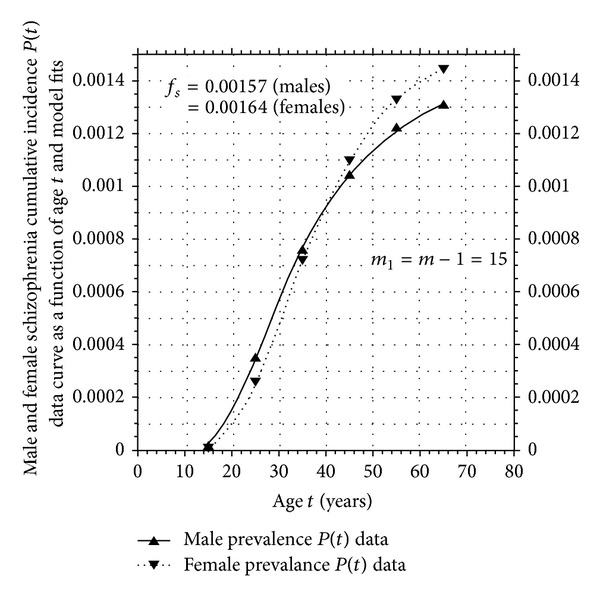
USAMF4P. Four-parameter independent mutation model fit to Kramer male and female USA schizophrenia first hospital admission cumulative incidence rate per 100,000 data.

The male and female cumulative incidence data as a function of age curves (i.e., the original data in [7]) as well as the 4-parameter model fits to them using (5) are plotted in Figure 1. The values of the male and female fit parameters appear in Table 1 and satisfy the modeling requirement that the number of steps or mutations *m* necessary to cause the onset of the disease (in analogy to uranium-238 decay into lead-203 in steps or stages) is independent of sex. In nuclear physics, the number of internal changes in a radioactive nucleus leading up to its spontaneous decay is unknown and factored into the measured value of its lifetime. In the same way, internal changes to the brain leading up to a schizophrenia mutation are ignored in this modeling, but it presumed that these changes or mutations could be observable with current or future neuroscience techniques. The values of the lifetime risk returned by these two fits refer to hospital admissions only. Assuming that the USA hospital admission cohort is a perfect random sample of the USA risk population as a whole, to get the actual schizophrenia prevalence of the entire USA risk population, we need only to replace the value of the *f*_*s*_ returned by the fit by the total USA value for the lifetime risk obtained by accurate survey data (*f*_*s*_ ≈ 0.01 or about 1%). This assumption will be used in all the modeling that follows. Thus, in this model, about 99% of the USA population cannot develop schizophrenia and can be regarded as unsusceptible to it. Since *m*_1_ = 15, of the 16 changes or mutations necessary to cause schizophrenia in this model, 15 schizophrenia mutations take place at the rate of *k*_1_, while the remaining one occurs at the rate *k*_2_. Thus, for the USA male risk population, the mean time for a schizophrenia mutation associated with *k*_1_ to occur is *T*_1_ = 1/*k*_1_ = 8.58 years, while the mean time for a schizophrenia mutation associated with *k*_2_ is *T*_2_ = 1/*k*_2_ = 35.1 years ≫*T*_1_. The analogous results for USA females are *T*_1_ = 10.1 years and *T*_2_ = 30.0 years, differing by modest 17.7% and −14.5% from the respective male results. However, the difference in the values of male and female lifetime risk *f*_*s*_ returned by the fits was only about 4%, suggesting that the prevalence of schizophrenia in the general USA population is largely independent of sex. The parameters for the fit to the aggregate male plus female USA data also appear in Table 1. 

**Figure 2:**
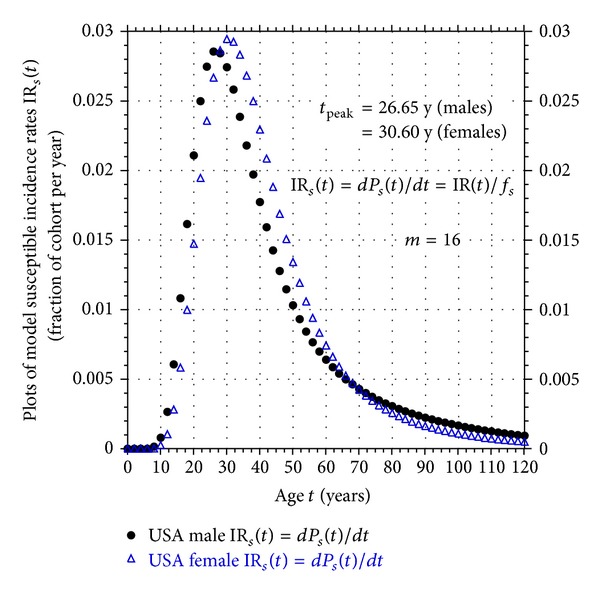
Plots of model susceptible incidence rates IR_*s*_(*t*) obtained from fits to USA male and female schizophrenia age-of-onset data.

Using the values of the parameters *m*, *k*_1_, and *k*_2_ returned by the fits, the male and female susceptible incidence rate curves IR_*s*_(*t*) computed from (4) and (5) are shown in Figure 2. As seen from this figure, the peak in the incidence rate curve for USA males occurs at the age of *t*_peak_ = 26.65 years, while the female curve peaks at *t*_peak_ = 30.60 years. These results are consistent with the known delayed onset of schizophrenia in females when compared to males [18].

The 3-parameter model fit to the USA male data also yields a credible fit but with a modest increase in fit error as seen in Table 1. Since we have found that the 4-parameter model always yields the best fit to data, only the 4-parameter model results will be presented from now on. Since most schizophrenia age-of-onset data are significantly imprecise due to biases, such as who got hospitalized, there is presumably some noise inherent in the data. The need to introduce a second mutation rate to accurately fit the data may be entirely due to noise. Thus, it is possible that if the data were perfect, then only one mutation rate *k* would be necessary to give excellent fits. 

## 3. Modeling Schizophrenia Twin Study Data

In this section, the schizophrenia MMM constructed in Section 2.1 for singleton cases will be extended to describe twin births. In a collection of monozygotic twins where one of the twins is schizophrenic, every member of the cohort is born with a susceptibility to develop schizophrenia, and so the risk fraction or lifetime risk is *f*_*s*_ = 1. In the analysis of all such studies, each twin pair must be separated and randomly assigned to two different subcohorts using a criterion that has nothing to do with schizophrenia, for example, by the random flipping of a coin. Thus, two subcohorts are assembled with identical twin pairs assigned to different subcohorts in random fashion. Thus, the age-of-onset prevalence curve *P*_*s*_(*t*) of the two subcohorts should be identical even though this function may have nothing to do with the model prevalence function given in Section 2 and has a form completely different from that in (5). This is one of the crucial tests of the validity of the model. Since the published twin studies contain no such analysis, it is essential to reanalyze the data in these studies to test this age-of-onset prediction. 

In our model of schizophrenia susceptibility, all members of both subcohorts are born with the susceptibility to develop the disease; thus, it is predicted that all monozygote cotwins will eventually develop schizophrenia if they can live long enough. 

### 3.1. Extending the Singleton Multimutation Model to Describe Twin Age-of-Onset Data

When one twin (the index twin) in each pair has developed the disease, the other twin will be referred to as the cotwin in this paper. Our model posits that in monozygote twin pairs, the cotwin has the same susceptibility to develop schizophrenia as the index twin. Consider a *birth* cohort of monozygotic twins all having the same age *t*. Assuming that birth is coincident with age *t* = 0, one twin (either the first or second born) will experience the onset of schizophrenia, say at age *t*. As soon as that happens, one twin is randomly assigned to subcohort 1 and the other to subcohort 2. All schizophrenia twin studies can easily assemble subcohorts 1 and 2 in this way. As these subcohorts age, their cotwins start experiencing the onset of schizophrenia. The risk of developing the disease at age *t* by members of a subcohort is given by a *susceptible* prevalence function *P*_*s*_(*t*), which in our modeling is defined in (5). Assuming that genetic factors are entirely responsible for the development of schizophrenia for any monozygote cohort, both susceptible subcohorts will experience the same susceptible prevalence function *P*_*s*_(*t*). Thus, when a member of subcohort 1 experiences the onset of schizophrenia at age *t*, the probability that the cotwin in subcohort 2 will [will not] develop the disease by this age is given by *P*_*s*_(*t*)[*Q*_*x*_(*t*) ≡ 1 − *P*_*s*_(*t*)]. 

The probability that any member of subcohort 1 will be found [will not to be found] to have schizophrenia by age *t* will be denoted by *P*_*s*_^(1)^(*t*)[*Q*_*x*_^(1)^(*t*) ≡ 1 − *P*_*s*_^(1)^(*t*)], with a similar notation for subcohort 2. Since *P*_*s*_^(*i*)^(*t*) + *Q*_*x*_^(*i*)^(*t*) = 1 for *i* = 1,2, we have

(7a) 1=[Ps(1)(t)+Qx(1)(t)][Ps(2)(t)+Qx(2)(t)]=Ps(1)(t)Ps(2)(t)+(Ps(1)(t)Qx(2)(t)+Ps(2)(t)Qx(1)(t))+Qx(1)(t)Qx(2)(t).

Thus, we define subcohort concordant, discordant, and nonschizophrenia probabilities as

(7b) $$\begin{array}{c} P_{s,s}\left(t\right)\equiv P_{s}^{\left(1\right)}\left(t\right)P_{s}^{\left(2\right)}\left(t\right),\\ P_{s,x}\left(t\right)\equiv \left(P_{s}^{\left(1\right)}\left(t\right)Q_{x}^{\left(2\right)}\left(t\right)+P_{s}^{\left(2\right)}\left(t\right)Q_{x}^{\left(1\right)}\left(t\right)\right),\\ P_{x,x}\left(t\right)\equiv Q_{x}^{\left(1\right)}\left(t\right)Q_{x}^{\left(2\right)}\left(t\right), \end{array}$$

respectively, where

(7c) $$\begin{array}{c} P_{s,s}\left(t\right)+P_{s,x}\left(t\right)+P_{x,x}\left(t\right)=1. \end{array}$$

It is important to note that subcohort concordance as defined above, for example, is not the same as pairwise concordance as usually used in the literature. Here, if a member of subcohort 1 and a member of subcohort 2 are chosen at random at age *t*, the probability that *both* will have acquired schizophrenia is given by *P*_*s*,*s*_(*t*), and the probability that they will be found to be discordant is denoted by *P*_*s*,*x*_(*t*). Finally, the quantity *P*_*x*,*x*_(*t*) is the probability that *neither* one of them will be found to be schizophrenic at age *t* even though they are both susceptible to developing the disease. 

For monozygote (MZ) twins, subcohorts 1 and 2 are genetically identical so that *P*_*s*_^(1)^(*t*) = *P*_*s*_^(2)^(*t*) ≡ *P*_*s*_(*t*) and *Q*_*s*_^(1)^(*t*) = *Q*_*s*_^(2)^(*t*) ≡ *Q*_*s*_(*t*); thus, the probabilities in (7b) become

(8) Ps,s(t)=Ps2(t),Ps,x(t)=2Ps(t)Qs(t),Px,x(t)=Qx2(t),   [MZ  twins].

When a susceptible monozygote twin pair in the (*x*, *x*) state (neither twin has developed schizophrenia yet) makes a transition to the (*s*, *x*) state at age *t*, it means that one of the twins has developed schizophrenia at age *t* (the age-of-onset). The probability that such a transition would take place, denoted by *dP*_*s*,*x*_^+^(*t*), is given by

(9a) $$\begin{array}{c} dP_{s,x}^{+}\left(t\right)=-dP_{x,x}\left(t\right). \end{array}$$

Integrating this result from *t* = 0 to any age *t* gives

(9b) Ps,x+(t)=1−Px,x(t)=1−[1−Ps(t)]2=Ps(t)[2−Ps(t)]

since *P*_*s*,*x*_^+^(0) = 0 and *P*_*x*,*x*_(0) = 1. Since *P*_*s*,*x*_^+^(*t*) is the age-of-onset distribution curve for the first twin of a pair that is susceptible to developing schizophrenia, the result in (9b) is extremely important in describing monozygote discordance. Notice that although it might have been expected that *P*_*s*,*x*_^+^(*t*) would turn out to be equal to *P*_*s*_(*t*), as it is in single-births, (9b) for twins shows that this is not true. It is also very important to note that the prevalence function *P*_*s*_(*t*) in this section is completely independent of the mutation model version of this function constructed in Section 2.1 above.

In schizophrenia twin studies, the birth cohort consists of only the concordant and discordant twin cases since, to date, it remains difficult to determine susceptibility to schizophrenia unless the disease is emerging (as in some prodrome cases) or actually develops. Thus, referring back to the results in (8), the fraction *C*_*ss*_^*M*^ of the monozygote birth cohort that is concordant at age *t* is given by

(10) CssM(t)≡Ps,s(t)[Ps,s(t)+Ps,x(t)]=Ps2(t)[Ps2(t)+2Ps(t)Qs(t)]=Ps(t)[Ps(t)+2(1−Ps(t))]

or

(11a) $$\begin{array}{c} C_{M}\left(t\right)\equiv C_{ss}^{M}\left(t\right)\equiv \frac{P_{s,s}\left(t\right)}{\left[P_{s,s}\left(t\right)+P_{s,x}\left(t\right)\right]}=\frac{P_{s}\left(t\right)}{\left[2-P_{s}\left(t\right)\right]}. \end{array}$$

Notice that the monozygote concordance rate *C*_*M*_(*t*) ≡ *C*_*ss*_^*M*^(*t*) is a function of *P*_*s*_(*t*). Since *P*_*s*_(0) = 0 and *P*_*s*_(*∞*) = 1, the monozygote concordance rate also varies between 0 and 1. Inverting (11a) by solving for *P*_*s*_(*t*) gives

(11b) $$\begin{array}{c} P_{s}\left(t\right)=\frac{2C_{M}\left(t\right)}{\left[1+C_{M}\left(t\right)\right]},\;\left[\text{Monozygotic}\;\;\text{twins}\right]. \end{array}$$

Since the value of *C*_*M*_(*t*) is determined from twin studies, the result in (11b) is a model prediction of the value of *P*_*s*_(*t*); this prediction can be tested by reanalyzing the data in the twin studies to compute this quantity. 

For the dizygotic twin cases, the formal results in (7a), (7b), and (7c) carry over here. Keeping the superscript (1) to refer to the schizophrenic index twin and superscript (2) to refer to the fraternal cotwin, a new expression for *P*_*s*_^(2)^(*t*) must be developed. To this end, we define the probability that a fraternal cotwin of a schizophrenic will also inherit the susceptibility to develop schizophrenia and denote this probability by *S*_inher_. Then, we can set

(12a) Ps(2)(t)=SinherPs(1)(t)≡SinherPs(t),where  Qs(2)(t)=1−Ps(t)  as  before.

Using (12a) in (7a), (7b), and (7c) then gives, analogous to (8),

(12b) $$\begin{array}{c} P_{s,s}\left(t\right)=S_{\text{inher}}P_{s}^{2}\left(t\right), \end{array}$$

(12c) Ps,x(t)=Ps(t)[1−SinherPs(t)]+[1−Ps(t)]SinherPs(t)=[1+Sinher]Ps(t)−2SinherPs2(t)

for dizygotic twins.

In the same way, the fraction *C*_*ss*_^*D*^(*t*) of the dizygote birth cohort that is concordant at age *t* is given by

(13a) CD(t)≡CssD(t)≡Ps,s(t)[Ps,s(t)+Ps,x(t)]=SinherPs(t)[1+Sinher−SinherPs(t)].

Notice that the dizygote concordance rate *C*_*D*_(*t*) ≡ *C*_*ss*_^*D*^(*t*) is also a function of *P*_*s*_(*t*). Since *P*_*s*_(0) = 0 and *P*_*s*_(*∞*) = 1, the dizygote concordance rate varies between 0 and *S*_inher_. 

Solving (13a) for the unknown probability *S*_inher_ gives

(13b) $$\begin{array}{c} S_{\text{inher}}=\frac{C_{D}\left(t\right)}{\left[P_{s}\left(t\right)+\left(P_{s}\left(t\right)-1\right)C_{D}\left(t\right)\right]}. \end{array}$$

Using (11b) in (13b) gives

(13c) $$\begin{array}{c} S_{\text{inher}}=\frac{\left[1+C_{M}\left(t\right)\right]}{\left[2C_{M}\left(t\right)/C_{D}\left(t\right)-1+C_{M}\left(t\right)\right]}. \end{array}$$

The values of the monozygote and dizygote concordance fractions *C*_*M*_(*t*) and *C*_*D*_(*t*), respectively, are determined by twin studies, so (13c) is a model prediction of the value of *S*_inher_, the probability that a fraternal cotwin of a schizophrenic will also inherit the susceptibility to develop schizophrenia. This prediction of the model can be tested by reanalyzing the data in classical twin studies to compute the value of *S*_inher_. It is again important to note that the prevalence function *P*_*s*_(*t*) in this section is independent of the mutation model version of this function; thus, all of the formulas from (7a) to (13c) are independent of any model.

### 3.2. Results of Modeling Age-of-Onset of Schizophrenia Twin Data

**Figure 3:**
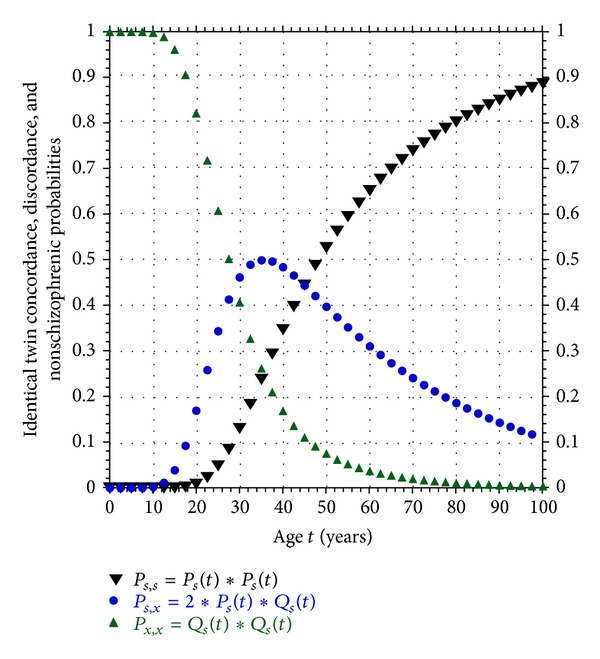
Plots of concordance probability *P*_*s*,*s*_(*t*), discordance probability *P*_*s*,*x*_(*t*), and nonschizophrenic probability *P*_*x*,*x*_(*t*) for identical twins susceptible to developing schizophrenia using the USA male plus female *P*_*s*_(*t*) curve.

Using the singleton USA male plus female multimutation model, the probability curves for identical twin concordance, discordance, and no-schizophrenia defined in (8), respectively, are plotted in Figure 3. Notice that the concordance probability curve *P*_*s*,*s*_(*t*) monotonically increases with age but never reaches saturation at 100% during a normal lifetime. In fact, even at the age of 80 years old, *P*_*s*,*s*_(*t*) ≈ 0.8, *P*_*s*,*x*_(*t*) ≈ 0.2, *P*_*x*,*x*_(*t*) ≈ 0, so that there is only an 80% chance that both members of a susceptible monozygote pair will have developed schizophrenia, and a 20% chance that they will be discordant. 

What twin studies actually measure is the *average* value of concordance for a cohort made up of members with a variety of different ages. Suppose that the cohort ranges from a low age *t*_*L*_ to a high age *t*_*H*_, where *t*_*H*_ − *t*_*L*_ = *b* years. Let *t*_*a*_ denote the age of a member of the cohort in years, where the index *a* = 1,2,…, *b* and where *t*_1_ = *t*_*L*_ and *t*_*b*_ = *t*_*H*_. If *n*_*a*_(*t*_*a*_) denotes the number of members of the cohort with age *t*_*a*_ and if the total number of members of the cohort is *N*_*T*_, then the average monozygote concordance measured for the cohort is given by

(14a) $$\begin{array}{c} \langle C_{M}\left(t\right)\rangle =\sum_{a=1}^{b}C_{M}\left(t_{a}\right)\frac{n_{a}\left(t_{a}\right)}{N_{T}}. \end{array}$$

For a uniform distribution of ages where *n*_*a*_(*t*_*a*_) is a constant, independent of age *t*_*a*_, the result in (14a) reduces to

(14b) $$\begin{array}{c} \langle C_{M}\left(t\right)\rangle =\frac{\int_{t_{L}}^{t_{H}}C_{M}\left(t\right)dt}{t_{H}-t_{L}},\;\left(\text{Uniform}\;\;\text{distribution}\right). \end{array}$$

Similar expressions apply for the average dizygote concordance 〈*C*_*D*_(*t*)〉.

The average monozygote and dizygote concordance rates from representative samples of schizophrenia twin studies from around the world are summarized in Table 2 [17, 19]. Only significant studies published before 1970 are included here in the hope that follow-up studies of the reported discordant twins would be carried out to definitively support or refute the predictions of the model. Virtually, none of these studies published either the age or schizophrenia age-of-onset distributions of their twin cohort, so the following analysis will make due without these data. In what follows, we shall show that the monozygote and dizygote concordance rates computed in these studies can be reproduced by our USA singleton multimutation model prevalence function by considering the results of three studies shown in Table 2. 

Let us first consider the Gottesman and Shields data [14, 15]. The age range for the Gottesman cohort in Table 2 was reported to be 19 y < *t* < 64 y, with the median age being 37 y [20]. Let us assume that the schizophrenia age-of-onset distribution curve for the Gottesman cohort is identical to that of the USA modeled in Section 2.2. Then, the average monozygote concordance rate for the Gottesman cohort must fall within the range from *C*_*M*_(19 y) = 0.0278 to *C*_*M*_(64 y) = 0.724, which it clearly does. Assuming that the Gottesman cohort is close to a uniform distribution, then (14b) yields an average monozygote concordance value of 〈*C*_*M*_(*t*)〉 = 0.4012, very close to the value of 10/24 = 0.4166 obtained by Gottesman. Since a USA age cohort of age *t* = 41 years has a monozygote concordance of 10/24, the Gottesman cohort is equivalent to a USA age cohort of 41 years old. The values of *P*_*s*_(41 y) = 0.588 and *S*_inher_ = 0.165 shown in Table 2 are computed using a USA age cohort that is 41 years old. Notice that two other studies in Table 2 [13, 16] have data very close to that of Gottesman and, therefore, these cohorts are also very likely described by the USA age-of-onset distribution curve.

We next turn our attention to the Hoffer and Pollin [17] results shown in Table 2. The Hoffer and Pollin study was composed of 15,930 US military twin pairs where both twins served in the armed forces. Since all members accepted into the USA military had to pass a rigorous mental-health exam, it is very likely that many potential recruits at risk for schizophrenia were rejected, and skewed results can therefore be expected from this study. The age range for this cohort was 38 y < *t* < 48 y, so this cohort is very close to being an age cohort with an average age of 43 years old. The average monozygote concordance measured by Hoffer and Pollin was 11/80 = 0.137. In the USA age cohort model, a monozygote concordance of this value occurs around 16.5 years of age, far *below* the age range of this cohort. Thus, the Hoffer and Pollin and USA age-of-onset distribution curves must be radically different. Nonetheless, we shall show that both sets of data can be described by the same risk function *P*_*s*_(*t*) but with different values for the mutation rate parameters.

As we have seen in Section 2.2, the USA prevalence function for an age cohort with age *t* is given by

(15a) $$\begin{array}{c} P_{s}\left(t\right)\equiv P_{s}\left(t;k_{1},k_{2}\right)={\left[1-\exp\left(-k_{1}t\right)\right]}^{15}\left[1-\exp\left(-k_{2}t\right)\right], \end{array}$$

where *k*_1_ = 0.10757 y^−1^ and *k*_2_ = 0.029959 y^−1^. To represent the Hoffer and Pollin prevalence function, we will assume that it has a modified version of the result in (15a), namely,

(15b) Ps(t,r)≡Ps(t;r·k1,r·k2)=[1−exp⁡(−rk1t)]15[1−exp⁡(−rk2t)],

where *r* is a dimensionless scaling factor that slows down (*r* < 1) or speeds up (*r* > 1) the rate at which schizophrenic mutations occur (the biological clock rate). In this notation, *P*_*s*_(*t*, 1) ≡ *P*_*s*_(*t*; *k*_1_, *k*_2_) given in (15a). Using (11b), we find that, for an age cohort of *t* = 43 y, (15b) must satisfy

(16a) Ps(43 y;r)=[1−exp⁡(−43rk1)]15[1−exp⁡(−43rk2)]=2291=0.2417.

**Figure 4:**
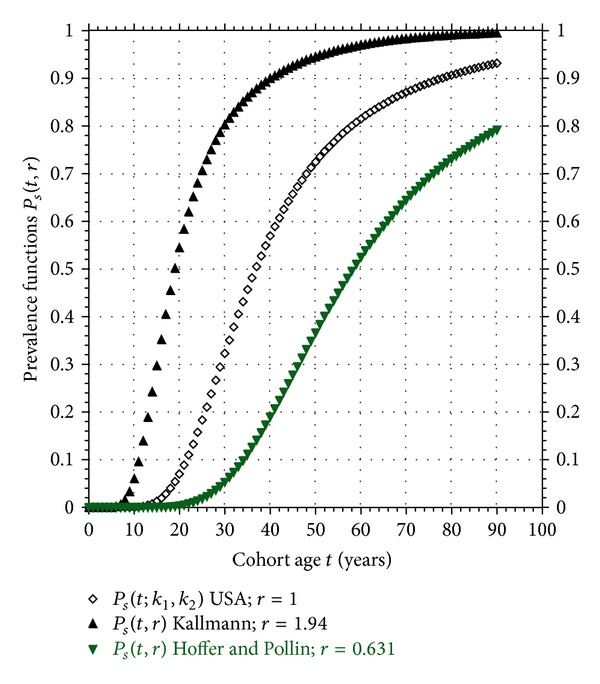
Comparison of schizophrenia prevalence functions *P*_*s*_(*t*, *r*) for USA data (*r* = 1), Hoffer and Pollin twin cohort (*r* = 0.631), and Kallmann twin cohort (*r* = 1.94).

Numerically solving (16a) yields the value

(16b) r=0.631, (Hoffer  and  Pollin)

a result that was also placed in Table 2. Thus, the Hoffer and Pollin age-of-onset prevalence function has exactly the same form as that of the USA but with mutation rates *k*_1_′ = *rk*_1_and *k*_2_′ = *rk*_2_ in place of *k*_1_ and *k*_2_, respectively. A plot of the Hoffer and Pollin and USA prevalence functions appear in Figure 4. These curves differ in the value of only a single parameter—the biological clock rate parameter *r*.

As a final example, consider the largest twin study in Table 2, that of Kallmann [10]. From Table 2, the measured monozygote concordance rate in the Kallmann study is 120/174. Using this value in (11b), yields *P*_*s*_(*t*) = 0.816, where *t* is the average age of the concordant members of the cohort at this point. Using the singleton USA male plus female *P*_*s*_(*t*) curve that results from the parameters in Table 1, we predict that the age of the Kallmann cohort when this concordance rate was reached was *t* = 60.3 years old. However, since the Kallmann cohort ranged in age from a low of *t*_*L*_ = 15 years to a high of *t*_*H*_ = 45 years, the prediction from the USA data is above this range and, therefore, the USA prevalence function is inconsistent with the Kallmann data. Thus, the prevalence curves for the Kallmann and USA cohorts must be significantly different. We proceed here in the same way that we did in the Hoffer and Pollin analysis above. We now assume that the Kallmann prevalence function is given by (15b) where the parameter *r* must be determined from the data. To determine the value of *r*, we will use the expression for the mean age $\overset{-}{t}$ at which schizophrenia is developed in a cohort

(17) $$\begin{array}{c} \overset{-}{t}\equiv \frac{\int_{t_{L}}^{t_{H}}tdP_{s}\left(t,r\right)}{P_{s}\left(t_{H},r\right)-P_{s}\left(t_{L},r\right)}. \end{array}$$

Since $\overset{-}{t}=23.8$ y for the Kallmann cohort, (17) is an equation for *r*. Numerically solving this equation yields the solution *r* = 1.94, a result that was also placed in Table 2. It then remains to solve

(18) $$\begin{array}{c} P_{s}\left(t,r\right)={\left[1-\exp\left(-rk_{1}t\right)\right]}^{15}\left[1-\exp\left(-rk_{2}t\right)\right]=0.816, \end{array}$$

for the age *t* since *r*, *k*_1_ and *k*_2_ are known. The numerical solution to (18) is = 31.0 y, a value that is almost exactly in the middle of the age range for the Kallmann cohort. The Kallmann prevalence function in (18) is also plotted in Figure 4, and it is now apparent that the USA prevalence curve (*r* = 1) is, approximately, an average of the Hoffer and Pollin, Kallmann, and other prevalence curves in Table 2. Thus, not all cohorts have prevalence curves with the same dependence on age *t*, but if we average over all of them, we expect to get the USA result. Nonetheless, all of the prevalence curves have the same form shown in (15b), and, therefore, they are generated by the same multimutation model describing the development of schizophrenia with *m* = 16 mutations. The exceptionally large value for the Kallmann monozygote concordance rate (68.9%) may be traced to the fact that his cohort largely consisted of severe or chronic schizophrenics in hospitals catering to long-stay patients [19].

In the twin data analysis, we introduced the probability that a fraternal cotwin of a schizophrenic will also inherit the susceptibility to develop schizophrenia and denoted it by *S*_inher_ (see (12a)). Then, using the results in (13c) and (11b) in Section 3.1, the value for *S*_inher_ predicted by the modeling can be calculated, and the results also appear in Table 2. Table 2 also contains the predicted value of the schizophrenia prevalence *P*_*s*_(*t*, *r*) of a birth cohort at age *t* when the monozygote concordance reaches the value shown in this table. Since the values for these two quantities can be computed directly from the schizophrenia twin study data, the predictions for these quantities in Table 2 constitute tests of the model. 

Although the monozygote concordance rates of these studies vary widely (from 0.138 to 0.689), the range in the value of *S*_inher_ is found to be 0.116 < *S*_inher_ < 0.283 with the result of 0.129 for the Kallmann data being near the lower end of this range. Since the Kallmann study had the largest cohort of monozygote twin pairs by far, it is clearly the most important study in this table. The results of the Japanese study by Inouye produced the value of *S*_inher_ = 0.163, slightly above the Kallmann result. In fact, all but the result for the Kringlen study appearing in Table 2 produce values for *S*_inher_ that are within a factor of 2 of that obtained from the Kallmann data. Since the risk for schizophrenia in children with one schizophrenic parent is 16.4% (0.164) [21], the values of *S*_inher_ in the table average out to be about this value.

**Figure 5:**
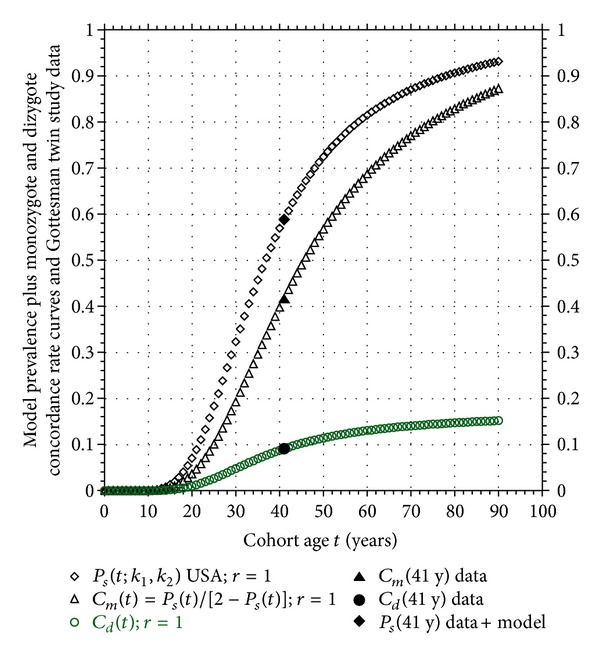
Plots of Gottesman and Shields prevalence *P*_*s*_(*t*), monozygote concordance *C*_*m*_(*t*), and dizygote concordance *C*_*d*_(*t*) curves together with corresponding twin study data from Table 2.

Using the Gottesman and Shields data as a typical example of the results we have obtained, the susceptible prevalence *P*_*s*_(*t*, *r* = 1) is plotted in Figure 5 (see (5) and Table 1). Using the same model in (11a), the monozygote concordance rate curve is also plotted in Figure 5. Finally, using the USA model coupled with the value of *S*_inher_ for the Gottesman and Shields study in Table 2, the dizygote concordance curve for the this twin cohort is plotted in Figure 5 using (13a). When the monozygote concordance of this cohort reaches the value of 10/24 = 0.416, the prevalence is *P*_*s*_(*t*, 1) = 0.588 at the age *t* = 41.0 years, and the dizygote concordance is *C*_*D*_(*t*) = 0.1025; all three of these points fall exactly on their respective curves in Figure 5. Changing the value of *S*_inher_ in (13a) to match the value of the different studies, we see that each study generates a dizygote concordance rate curve that has the same characteristic as the one plotted in Figure 5; namely, it plateaus at the maximum value of *S*_inher_ itself. If this model prediction of the plateauing of the dizygote concordance curve turns out to be correct, then it would support the proposition that the susceptibility to develop schizophrenia is acquired by internal, genetic factors, not external environmental ones. 

The model predicts that both the monozygote and dizygote concordance rate curves are monotonically increasing functions of age but saturate at 1 and *S*_inher_ ≪ 1, respectively, very different values, as seen in Figure 5. These predictions can easily be tested by revisiting the classical twin studies using *the same* cohorts and bringing the data up-to-date. 

We can find only one study that made one follow-up diagnosis of the nonill monozygote cotwins after variable years [22]. This study supports our proposition by showing increases in both concordance rate and new psychopathology among previously healthy cotwins, although the follow-up interval was not long enough, nor the age of the twins were old enough to provide quantitative support to the model. From the monozygote concordance rate curve that appears in Figure 5, note that 100% concordance is generally not possible to observe because, again, this value occurs at an age *t* above the maximum human life span, although recollecting twin data in their advanced age should provide sufficient test of the model. 

Now, the fraction of dizygotic cotwins that has susceptibility to develop schizophrenia is, by definition, *S*_inher_. Thus, the fraction of dizygotic twins that is unable to develop schizophrenia is 1 − *S*_inher_. Using the calculated values of *S*_inher_ shown in Table 2, we calculate that at least 71% [(1 − *S*_inher_) × 100%] of cotwins in dizygotic twin studies is predicted to be unable to develop schizophrenia even though the cotwin shared a similar environment as their schizophrenic twin. This prediction would not support substantial environmental (prenatal or postnatal) contribution to schizophrenia susceptibility. 

## 4. Conclusion

Although a wide variety of prenatal maternal infections, such as influenza, herpes, polio, rubella, and toxoplasmosis, have been linked to schizophrenia [23, 24], many investigations have shown that prenatal exposure to infection did not significantly increase the risk [25]. The data linking prenatal exposure to influenza and schizophrenia remain contradictory [26]. For example, in an investigation of psychiatric admissions of people born a few months after the 1957 A2 influenza epidemic in Scotland, it was found that only 3 children of the 945 born to mothers who actually suffered from influenza during the second trimester of pregnancy became schizophrenics; this risk rate was no greater than that faced by children of mothers who were not infected [27]. A study using Japanese government data reached the identical conclusion that there was no relationship between influenza epidemics and schizophrenic births [28]. Thus, the genetic multimutation model described here remains a viable explanation for very disparate data on schizophrenia. The multimutation model constructed here is shown to fit monozygote and dizygote concordance rate data of important twin studies completed before 1970 in addition to singleton age-of-onset data. Thus, revisiting the historical twin studies listed in Table 2 to reexamine the previously declared nonill cotwin's diagnostic status at their advanced age would be a test of this random multimutation model. 

## Figures and Tables

**Table 1:** Values of model parameters for the independent mutation model fits to USA schizophrenia first hospital admissions data (males M, females F, and males + females, M + F).

Cohort	Number of parameters in model	*m* mutation number	*k* or *k*_1_ mutation rate in (years)^−1^ [*m*_1_ = *m* − 1]	*k*_2_ mutation rate in (years)^−1^ [*m*_2_ = 1]	*f*_*s*_ lifetime risk	chisq error in (years)^−2^
USA males	3	10	0.08172		0.00137	9.22*e* − 10
USA males	4	16	0.11653	0.028465	0.0015737	3.53*e* − 10
USA females	4	16	0.09859	0.035728	0.0016428	7.82*e* − 11
USA males + females	4	16	0.10757	0.029959	0.0016363	2.26*e* − 10

**Table 2:** Concordance rate table. Uncorrected concordance rates in schizophrenia twin studies from around the world and modeling results from fits to these data. Only significant studies published before 1970 are included here so that the updates of these studies could definitively test the predictions of the model.

Investigator	Year	Country	MZ pairs concordance	DZ pairs concordance	*S*_inher_*	*P*_*s*_(*t*,*r*)^#^	*t*, (*r*)
Rosanoff et al. [8]	1934	USA	28/41 = 0.683	15/101 = 0.149	0.190	0.812	
Essen-M$\ddot{\text{o}}$ller[9]	1941	Sweden	6/11 = 0.545	4/27 = 0.148	0.224	0.706	
Kallmann [10]	1946	USA	120/174 = 0.689	53/517 = 0.102	0.129	0.816	31.0 y, (1.94)
Slater [11]	1953	UK	24/37 = 0.648	10/112 = 0.0892	0.116	0.787	
Inouye [12]	1961	Japan	33/55 = 0.600	2/17 = 0.117	0.163	0.750	
Harvald and Hauge [13]	1965	Denmark	4/9 = 0.444	6/62 = 0.0967	0.167	0.615	
Gottesman and Shields [14, 15]	1966	UK	10/24 = 0.416	3/33 = 0.0909	0.165	0.588	41.0 y, (1)
Kringlen [16]	1966	Norway	19/50 = 0.380	13/94 = 0.138	0.283	0.551	
Hoffer and Pollin [17]	1970	USA	11/80 = 0.137	6/145 = 0.0413	0.197	0.242	43 y, (0.631)

*S_inher_ is defined as the probability that a fraternal cotwin of a schizophrenic will also inherit the susceptibility to develop schizophrenia.

^#^ *P*_*s*_(*t*, *r*) is the prevalence obtained from a model simulation of twin study concordance results for a susceptible age cohort at age *t*.
